# Positron Beam Loading and Acceleration in the Blowout Regime of a Plasma Wakefield Accelerator

**DOI:** 10.34133/research.0878

**Published:** 2025-09-09

**Authors:** Shiyu Zhou, Siqin Ding, Weiming An, Qianqian Su, Jianfei Hua, Fei Li, Warren B. Mori, Chan Joshi, Wei Lu

**Affiliations:** ^1^ Institute of High Energy Physics, Chinese Academy of Sciences, Beijing 100049, China.; ^2^ Department of Engineering Physics, Tsinghua University, Beijing 100084, China.; ^3^ School of Physics and Astronomy, Beijing Normal University, Beijing 100875, China.; ^4^ Institute for Frontiers in Astronomy and Astrophysics, Beijing Normal University, Beijing 102206, China.; ^5^ University of California Los Angeles, Los Angeles, CA 90095, USA.

## Abstract

Plasma wakefield acceleration in the nonlinear blowout regime has achieved marked milestones in electron beam acceleration, demonstrating high acceleration gradients and energy efficiency while preserving excellent beam quality. However, this regime is deemed unsuitable for achieving positron acceleration of comparable results, which is vital for future compact electron–positron colliders. In this article, we find that an intense positron beam loaded at the back of beam-driven blowout cavity can self-consistently induce the focusing field and flatten the longitudinal wakefield, leading to stable, high-efficiency, and high-quality positron acceleration. This is achieved through the formation of an on-axis electron filament induced by positron beam load, which shapes the plasma wakefield in a distinct way compared to electron beam load in the blowout regime. Via a nonlinear analytic model and numerical simulations, we explain the novel beam loading effects of the interaction between the on-axis filament and the blowout cavity. High-fidelity simulations show that a high-charge positron beam can be accelerated with >20% energy transfer efficiency, ~1% energy spread, and ~1 mm·mrad normalized emittance, while considerably depleting the energy of the drive beam. The concept can also be extended to simultaneous acceleration of electron and positron beams and high transformer ratio positron acceleration as well. This development offers a new route for the application of plasma wakefield acceleration into particle physics.

## Introduction

The demand for particle physics at the energy frontier has motivated intense research on novel accelerator techniques with higher accelerating gradient and energy transfer efficiency. This is particularly so for future high-energy electron–positron colliders because leptons have an ever-increasing synchrotron energy loss as the beam energy increases [1–3]. Compared to the radio-frequency accelerators, plasma-based acceleration surpasses the breakdown limit and can support accelerating gradients several orders of magnitude larger [4,5]. Based on this remarkable progress over the past decades, several schemes have been proposed to incorporate plasma acceleration to future electron–positron colliders [6–8]. Within this context, a charged particle-driven plasma wakefield accelerator (PWFA) has made a series of breakthroughs in electron beam acceleration, which provides sustained acceleration gradients up to 50 GeV/m [9], energy extraction efficiencies of tens of percent [10], and energy spreads of ~1% [11] and maintains the excellent beam quality [12,13]. These groundbreaking results were obtained in the so-called blowout regime by using an ultrashort and high-peak-current electron beam to drive plasma electrons out of a roughly spherical region and leave behind a copropagating ion cavity that accelerates and focus the trailing electron beam [14–17]. However, comparable progress has remained elusive for positron beams—the essential counterparts to electrons in collider applications. This limitation arises mainly because of the region where the wakefield is both accelerating and focusing for positrons is extremely small and exists only when the blown-out plasma electrons are attracted by the ions to form a density spike [18–20].

Recent works are exploring the alternative solutions for the plasma wakefield positron acceleration. Several schemes in the hollow plasma channel using either a thin, warm channel or an asymmetric electron drive beam are exploited to accelerate the positron beam [21–23]. However, any misalignment between the drive/witness beams and the plasma channel center would lead to transverse instabilities [24]. An electron beam-driven wakefield in a plasma channel with finite width has been proposed for positron beam transport and acceleration, yet limited positron beam charge can be loaded in such a wake and the corresponding energy transfer efficiency from drive to witness beam is highly restricted [25,26]. Another approach utilizes an electron beam or laser driver with a doughnut shape to excite the plasma wake, allowing for on-axis focusing and acceleration of the positron beam [27,28]. However, the long-term propagation of such a driver may encounter azimuthal instability, jeopardizing the positron beam quality. In addition, while recent experiments have demonstrated the acceleration of the rear part of a positron beam in a self-loaded plasma wakefield, it is not applicable for multi-stage acceleration to achieve high beam energy [29]. Besides, these regimes utilize specific plasma structures or drive beams that have a customized profile that pose extra demands on the experimental setup and feasibility. Furthermore, there is still no theoretical model for the nonlinear positron beam loading effect and even a heuristic model is important for developing intuition, scaling laws, and beam quality optimization.

In this work, we propose a simple configuration for positron beam acceleration in the electron beam-driven blowout regime that can achieve stable, high-efficiency positron acceleration with great beam quality. We found that loading a positron beam just behind the first bubble in the blowout wake self-consistently induces an extended focusing region and a flattened accelerating field. In an unloaded blowout wake, sheath electrons between the end of the bubble (first bucket) and the front of the second bucket are transitioning from moving toward the axis to moving away. By placing an intense positron beam in this region, its space charge force attracts some sheath electrons back toward the axis forming an extended on-axis electron filament that overlaps with the positron beam. This filament provides the essential focusing force for the positron beam, while the unique interaction between this on-axis filament and the bubble sheath plasma electrons facilitates the flattening of the accelerating field. We extend the approach of Lu et al. [15,16] to develop the first theoretical model of nonlinear positron beam loading to include the interplay between the electron filament and the bubble profile. Three-dimensional (3D) quasi-static particle-in-cell (PIC) simulations show that it is able to simultaneously achieve ∼1% induced energy spread, emittance preservation of several micrometers, and energy transfer efficiency over 20% from wake to the positron beam. Furthermore, an additional electron bunch can be loaded in the same wake in the first bucket, which can lead to the improvement of the positron beam quality and overall beam loading efficiency. The possibilities of positron acceleration with a high transformer ratio (HTR) and even simultaneous HTR electron and positron acceleration with a single driver are also illustrated. The same idea can be applied to laser wakefield positron acceleration as well. These progresses pave the way for the plasma-based positron acceleration to be employed as a high-energy injector in future electron–positron colliders [6].

## Results

### The positron beam loading induced on-axis electron filament

**Fig. 1.**
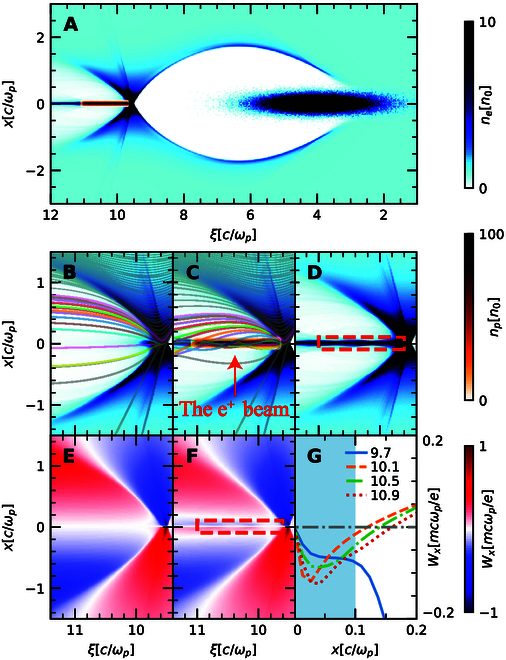
The plasma response and corresponding transverse wakefield in a positron beam loaded blowout regime. (A) Plasma and beam densities for a loaded blowout regime (see text below for the beam, plasma, and simulation parameters). The zoomed-in view and the trajectories of the plasma electrons for (B) the unloaded and (C and D) the positron beam loading case. Transverse wakefield $W_{x}\equiv E_{x}-cB_{y}$ for (E) the unloaded and (F) positron beam loading case and (G) its transverse lineouts at $k_{p}\xi =9.7,10.1,10.5,\text{and}\;10.9$, respectively. Red boxes indicate the position of the positron beam.

For the electron beam-driven positron acceleration, recent research has been exploiting schemes capable of providing both focusing and accelerating fields for the positron beam [21–28]. These regimes necessitate complicated plasma structure or beam profile, such as the hollow plasma channel or the donut-shaped electron beam. In contrast, within a bi-Gaussian beam-driven blowout regime within the uniform plasma, the same requirements can be satisfied through a positron beam loading. In the blowout regime of PWFA where the ions are uniformly distributed within the cavity, the transverse force for a relativistic positron particle ($v_{z}∼c$) is [13]

$$\begin{array}{c} F_{⊥e^{+}}\left(r\right)=e\left(E_{r}-v_{z}B_{\theta }\right)\\ \;=-e\frac{\partial \psi }{\partial r}=\frac{1}{2}r+\frac{1}{r}\int_{0}^{r}\left[\rho_{e}\left(r^{\prime }\right)-J_{\mathrm{ze}}\left(r^{\prime }\right)/c\right]r^{\prime }dr^{\prime } \end{array}$$ (1)

where $\psi \equiv \phi -A_{z}$ is the wake pseudo-potential, ϕ and A are the scalar and vector electromagnetic potential, $\rho_{e}$ and $J_{e}$ are the charge and current density of the plasma electrons, and axial symmetry and quasi-static approximation (QSA) [30] are assumed. Henceforth, we adopt normalized units with length, speed, density, mass, and charge normalized to the plasma skin depth, $c/\omega_{p}$, speed of light, c, plasma density, $n_{0}$, electron rest mass, m, and electron charge, e, respectively. In Eq. 1, the first term is the repulsive force from ions and the second term is focusing induced by the plasma electrons remaining within the cavity. To focus a positron beam within transverse size $r_{0}$,

$$-\int_{0}^{r_{0}}\left[\rho_{e}\left(r^{\prime }\right)-J_{\mathrm{ze}}\left(r^{\prime }\right)/c\right]r^{\prime }dr^{\prime }>\frac{1}{2}r_{0}^{2}$$ (2)

must be satisfied. In an unloaded blowout wake, only the narrow volume between the first and second bubbles fulfills the criterion as in Fig. 1B. However, placing a positron beam into this region dramatically affects the distribution of the plasma electrons around. The transverse force for a plasma electron with longitudinal speed $v_{z}$ is

$$\begin{array}{c} F_{⊥e^{-}}\left(r\right)=-\frac{r}{2}-\frac{1-v_{z}}{r}\int_{0}^{r}n_{b}r^{\prime }dr^{\prime }\\ \;-\frac{1}{r}\left[\int_{0}^{r}\rho_{e}r^{\prime }dr^{\prime }\right.-\left.v_{z}\int_{0}^{r}\frac{J_{\mathrm{ze}}}{c}r^{\prime }dr^{\prime }\right]-\frac{1-v_{z}}{2}\int_{0}^{r}\frac{\partial E_{z}}{\partial \xi }dr^{\prime } \end{array}$$ (3)

where $n_{b}$ is the witness positron beam density and ξ≡ct−z. The 4 terms that contribute to the transverse force are due to the ions, positron beam, $\rho_{e}$ and $J_{\mathrm{ze}}$, and the plasma transverse current, respectively.

The plasma electron densities and the corresponding transverse wakefield distributions in a typical blowout regime with/without positron beam loading are visualized in Fig. 1 based on PIC simulations and particle trackings. Figure 1A depicts the electron beam-driven blowout regime in the (x,ξ) plane where $n_{\text{driver}}\gg n_{0}$, and $k_{p}\sigma_{r,z}\leq 1$. The plasma electrons are repelled by the relativistic drive electrons and then pulled back by the exposed ions, forming a bubble void of electrons. In the unloaded situation as presented in Fig. 1B and E, most electrons are reflected due to self-repulsion force of the plasma electrons (the third and fourth terms in Eq. 3) at the end of the bubble [31]. As these electrons are deflected, the focusing force provided by the immobile plasma ions (and the positron beam if placed as in Fig. 1A) becomes dominant, which results in some plasma electrons bending and staying close to the axis in the second wake cavity to provide the focusing force for positrons. If the positron beam plus the ions balance the self-repulsion force of sufficient sheath electrons, the latter form a coaxial filament and the positron beam can be confined.

Simulations in this work are performed using the quasi-static 3D-PIC code QuickPIC [32,33], with the detailed configuration provided in Methods. The drive beam has a bi-Gaussian profile centered at $k_{p}\xi =4$ with $k_{p}\sigma_{z}=1$, $k_{p}\sigma_{r}=0.17$, and $n_{\text{driver}}/n_{0}=23$. If the plasma density is $7.8\times 10^{15}\;{\mathrm{cm}}^{-3},$ then $k_{p}^{-1}=60\;\mathrm{\mu m}$, corresponding to the drive beam containing 2.75 nC charge with $\sigma_{z}=60\;\mathrm{\mu m}$ and $\sigma_{r}=10\;\mathrm{\mu m}$. This driver excites a nonlinear blowout wake where the returning plasma electrons converge around the axis in a small volume. Figure 1E shows that in an unloaded case, the region of the electron convergence (density peak) is the only focusing area for an on-axis positron beam. However, if a narrow positron beam (in the simulation, it has a longitudinally flattop profile at $k_{p}\xi \in \left[9.6,11\right]$ and a transverse Gaussian profile of $\sigma_{p}=2\;\mathrm{\mu m}$ with a peak density 100$n_{0}$) is loaded into the cavity just behind the density peak as in Fig. 1A and C, the situation changes dramatically. We track the trajectories of plasma electrons whose original radii uniformly distribute between 0 and $1.5\;k_{p}^{-1}$. Electrons returning to the axis quickly diverge again in the unloaded situation (Fig. 1B), whereas the space charge force of the positron beam attracts many of these electrons (Fig. 1C) to the axis, thereby creating an extended electron filament that overlaps with the positron bunch (Fig. 1D). Figure 1F shows that now there is a net focusing force on the positron beam. The transverse lineouts in Fig. 1G validate that the entire positron beam ($\pm 3\sigma_{p}$) is focused. Unfortunately, since the electron density in the filament is nonuniform, the transverse focusing field experienced by the positrons is nonlinear along r and varies along ξ [22]. Emittance preservation in this situation can be achieved by approximate matching and slice-by-slice matching techniques [26,34]. In general, the self-consistent focusing force requires the narrow size and high intensity of the positron bunch. Otherwise, the positron beam may suffer from defocusing forces in some slices and thus diverge during long-term propagation.

### Nonlinear positron beam loading effects in the blowout regime

The highly nonlinear interaction between plasma electrons and beams poses a formidable challenge to the development of a theoretical model for plasma-based acceleration. In the blowout regime, the electron beam is loaded in a cavity void of plasma electrons; thus, the loaded wakefields can be quantified through the dynamics of the bubble boundary [35]. In contrast, positron acceleration presents extra complexity due to the spatial and temporal overlap between the positron beam and plasma electrons, which makes the interaction inherently nonlinear and far less tractable. To date, no theoretical framework has been established to describe these nonlinear positron beam loading effects. In this work, we propose the first theoretical model that captures the essential physics of positron loading in the blowout regime, offering both physical insight and practical guidance toward high-quality, high-efficiency positron acceleration.

When the positron beam is loaded at the head of the second bucket, an extended electron filament forms on the axis, creating a coaxial bubble. Upon applying the QSA [27], wakefields in PWFA are determined by the pseudo-potential ψ, which obeys the 2-dimensional Poisson equation

$$\nabla_{⊥}^{2}\psi =-\left(\rho -J_{z}/c\right).$$ (4)

**Fig. 2.**
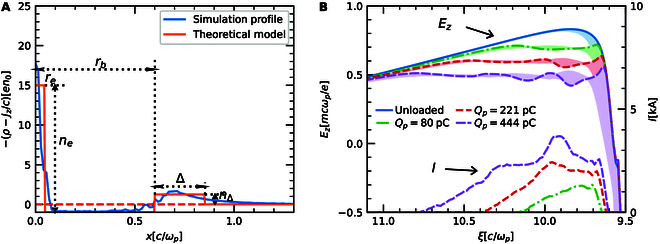
Beam loading effects in the coaxial bubble. (A) A transverse lineout of S at $k_{p}\xi =10.3$ in simulation and the approximation in theoretical model. (B) On-axis $E_{z}$ (line) and range of $E_{z}$ within $\pm 3\sigma_{p}$ (shadow) for different optimized positron currents.

Here, $\nabla_{⊥}\equiv \hat{x}\frac{\partial }{\partial x}+\hat{y}\frac{\partial }{\partial y}$ denotes the transverse gradient operator, and $\rho -J_{z}/c=\rho_{\mathrm{ion}}+\rho_{e}-J_{\mathrm{ze}}/c-J_{z,\mathrm{ion}}/c$ represents the difference between the charge and current densities. The source term can be defined as $S\equiv -\left(\rho -J_{z}/c\right)$, where $\rho_{\mathrm{ion}}=1$ and $J_{z,\mathrm{ion}}=0$ for immobile plasma ions. Figure 2A illustrates a lineout of S at $k_{p}\xi =10.3$ for the positron beam loading case in Fig. 1. In the coaxial blowout cavity, the source term S can be decomposed—proceeding from the axis outward—into contributions from the on-axis electron filament, the blowout region, the surrounding electron sheath, and the unperturbed background plasma, in a manner analogous to the conventional blowout regime [16,34]. Each component is approximated by a step-function profile of the source term S in our theoretical model, as illustrated in Fig. 2A.

The width and amplitude for the parts of the electron filament and electron sheath are $r_{e}$, $n_{e}$, Δ, and $n_{\Delta }$, respectively, and the blowout radius is $r_{b}$. With the piecewise-step approximation and the continuity of electric charge, the on-axis pseudo-potential, $\psi_{0}\equiv \psi \left(r=0\right)$, can be obtained by solving Eq. 4,

$$\psi_{0}\left(\xi \right)=\frac{1}{4}r_{b}^{2}\left(\xi \right)\left(1+\beta \left(\xi \right)\right)-\frac{1}{4}n_{e}\left(\xi \right)r_{e}^{2}\left(\xi \right)\left(1+\beta \left(\xi \right)\right)-\frac{1}{2}n_{e}\left(\xi \right)r_{e}^{2}\left(\xi \right)\text{ln}\frac{r_{b}\left(\xi \right)}{r_{e}\left(\xi \right)}$$ (5)

where $\beta \left(\xi \right)\equiv \frac{{\left(1+\alpha \right)}^{2}\ln{\left(1+\alpha \right)}^{2}}{{\left(1+\alpha \right)}^{2}-1}-1$ and $\alpha \equiv \Delta /r_{b}$. Without the electron filament ($n_{e}=0$ or $r_{e}=0$), we recover $\psi_{0}=\frac{1}{4}r_{b}^{2}\left(1+\beta \right)$ for a normal blowout regime [15,16]. When the electron filament is uniform along ξ ($\frac{\partial r_{e}}{\partial \xi }=\frac{\partial n_{e}}{\partial \xi }=0$) and assume a constant α as per convention, the on-axis $E_{z}$ can then be written as the summation of 2 terms, $E_{z1}$ and $E_{z2}$,

$$E_{z0}\left(\xi \right)=\frac{\partial \psi_{0}\left(\xi \right)}{\partial \xi }\equiv E_{z1}+E_{z2}=\frac{\partial }{\partial \xi }\left[\frac{r_{b}^{2}\left(\xi \right)}{4}\left(1+\beta \left(\xi \right)\right)\right]+\left[-\frac{1}{2}n_{e}r_{e}^{2}\frac{r_{b}^{\prime }\left(\xi \right)}{r_{b}\left(\xi \right)}\right]$$ (6)

In Eq. 6, $E_{z1}$ is determined by the shape of the bubble cavity just like in the case of electron beam loading [35], and $E_{z2}$ is a novel effect related to both the electron filament and the bubble profile. For an unloaded case, the longitudinal wakefield $E_{z}$ at the front of the second bubble is dominated by the first term $E_{z1}$, which decreases with ξ as in Fig. 2B. In the nonlinear blowout regime, this term is roughly proportional to $\frac{1}{2}r_{b}r_{b}^{\prime }$. When the positron beam is loaded in the region, the space charge force of the positrons decreases both $r_{b}$ and $r_{b}^{\prime }$ and thus more rapidly decrease $E_{z1}$. This means that the original theory of blowout regime indicates that the positron beam loaded in the cavity could not obtain uniform acceleration. However, the electron filament modifies the beam load effect in 2 ways. First, its space charge will cancel that of the positron beam and thereby decrease the effect on $r_{b}$. Second, $E_{z2}$ has the opposite sign with $E_{z1}$ and it also decreases in magnitude as ξ increases since $1/r_{b}$ and $r_{b}^{\prime }$ decrease as the bubble expands, which is exactly what is needed to flatten the wakefield.

On the other hand, plasma electrons are highly mobile and execute betatron oscillations due to the strong focusing force provided by the $e^{+}$ beam, which cause longitudinal variations in the filament. According to the Panofsky-Wenzel theorem,

$$\frac{\partial E_{z}\left(r,\xi \right)}{\partial r}=\frac{\partial W_{⊥}\left(r,\xi \right)}{\partial \xi }=\frac{\partial \psi \left(r,\xi \right)}{\partial r\partial \xi }=\frac{\partial }{\partial \xi }\left[-\frac{1}{r}\int_{0}^{r}\left[\rho_{e}\left(r^{\prime },\xi \right)-J_{\mathrm{ze}}\left(r^{\prime },\xi \right)/c\right]r^{\prime }dr^{\prime }\right],$$ (7)

longitudinal variations of the filament density (focusing force) lead to the transverse variation of $E_{z}$. This is substantially different from electron acceleration in the regular blowout regime, where the witness beam is located in the ion cavity devoid of plasma electrons. The highly immobile ions cause $\frac{\partial \psi \left(r,\xi \right)}{\partial r\partial \xi }$ to be negligible inside the cavity, allowing electrons at different transverse positions to experience the same $E_{z}$. For the positron beam acceleration in a coaxial cavity, the nonuniform $E_{z}$ makes positrons in the same longitudinal slice experience different accelerating fields, resulting in the average acceleration gradient being mismatched with the on-axis electric field.

Thus, a uniform average accelerating field $\left\langle E_{z}\right\rangle$, defined as $\frac{\int_{0}^{\infty }E_{z}\left(r\right)n_{b}\left(r\right)\mathrm{rdr}}{\int_{0}^{\infty }n_{b}\left(r\right)\mathrm{rdr}}$, along the positron beam is essential for beam quality optimization. Different cases of positron loading are presented in Fig. 2, where the parameters of the drive electron beam and plasma are consistent with those in Fig. 1. An iterative algorithm, detailed in Methods, is employed to optimize the beam current profile for uniform positron acceleration. Here, the positron beam has a Gaussian transverse profile with $\sigma_{p}=2\;\mathrm{\mu m}$, and both the drive and witness beams are assumed to be nonevolving. For different target accelerating fields, the corresponding current profiles and field distributions are displayed in Fig. 2B. To better describe the accelerating field experienced by the $e^{+}$ beam, the on-axis electric field (dashed line) and the range of $E_{z}$ within $\pm \;3\sigma_{p}$ (shaded area) are also shown. To extract more energy from the plasma wake, a higher charge is needed and the loaded $E_{z}$ becomes lower. However, owing to the nonlinear response of plasma electrons, the optimized shapes of the $e^{+}$ beam current profile are much different and the transverse variation of $E_{z}$ increases with the loaded charge because of the more intense effects described by Eq. 8. As a result, for the 80, 221, and 444 pC $e^{+}$ beams, $\langle E_{z}\rangle$ is 0.7, 0.6, and 0.5 $\mathrm{mc}\omega_{p}/e$, respectively, while the induced root mean square (rms) energy spread $\sigma_{\gamma }/\left(\gamma -\gamma_{0}\right)$ is 1.66%, 3.30%, and 4.56%, which indicates a trade-off between the beam charge, efficiency, gradient, and beam quality for positron acceleration in the blowout regime [36].

### Stable, high-efficiency, uniform positron acceleration schemes

The aforementioned theoretical analysis and numerical simulations elucidate the unique characteristics of positron beam loading in the blowout regime, revealing novel phenomena distinct from electron beam loading. These insights highlight the strong potential of the blowout regime for achieving high-quality positron acceleration. Based on this foundation, we demonstrate, through high-fidelity PIC simulations, a stable, high-efficiency, and high-quality positron acceleration sustained over long propagation distances.

**Fig. 3.**
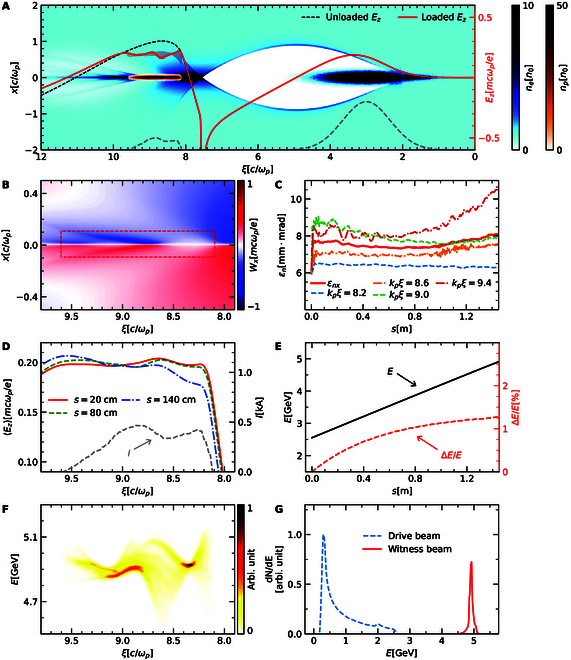
Results of the high-efficiency, high-quality positron beam acceleration in the blowout regime. (A) A snapshot of the plasma wake and beam densities after propagating 20 cm. The on-axis $E_{z}$ for the unloaded and positron beam load case are plotted with the red shadow denoting the range of loaded $E_{z}$ within $\pm 3\sigma_{p}$ in transverse dimension. (B) The distribution of transverse wakefield $W_{x}$, with the rectangle representing the position of positron beam. (C) Evolution of the slice and projected normalized emittances of the positron beam. (D) Averaged accelerating field $\langle E_{z}\rangle$ at the positron beam loaded phase for different propagation distances. (E) Evolution of the mean energy and induced energy spread. (F) Final longitudinal phasespace. (G) Final spectra of the drive and witness beams. The gray dashed lines in (A) and (D) denote the beam current profile.

In Fig. 3, we present a long-distance propagation case, where a bi-Gaussian electron beam with a charge of 534 pC, longitudinal and transverse sizes of $\sigma_{z}=40\;\mathrm{\mu m}$ and $\sigma_{r}=5\;\mathrm{\mu m}$, respectively, drives a plasma bubble in a background density of $n_{0}=7.8\times 10^{15}cm^{-3}$. The resulting blowout cavity reaches a maximum radius on the order of $k_{p}^{-1}$. This drive beam has a matched normalized emittance 20.8 mm·mrad for the main body and a lower emittance 0.5 mm·mrad for the head ($k_{p}\xi <2$) that suppresses the evolution of the beam profile. A positron beam with 98 pC of charge, transverse size $\sigma_{p}=2\;\mathrm{\mu m}$, and normalized emittance $\epsilon_{n}=6\;\mathrm{mm}\cdot \text{mrad}$ is loaded behind the bubble. Both beams have the initial energy of 2.5 GeV ($\gamma_{0}=5,000$).

Figure 3A illustrates a snapshot of the plasma profile, beam densities, and the corresponding $E_{z}$ at a propagation distance of 20 cm. The current profile of the $e^{+}$ beam is optimized as presented by the gray dashed lines in Fig. 3A and D such that the loaded $E_{z}$ is flattened, and the transverse variation of $E_{z}$ is also suppressed. The corresponding transverse wakefield in Fig. 3B shows that the entire $e^{+}$ beam (indicated by the dashed rectangle) is located in a region of focusing fields that varies along ξ as expected. Consequently, beam emittances at different longitudinal position evolve differently but share the same trend as plotted in Fig. 3C, which rapidly grow at the beginning then saturate at various levels. After propagation of around 100 cm, the emittances slowly increase again mainly because of the evolution of the drive beam. Finally, the projected emittance for the positron beam grows to about 8.1 mm·mrad at s= 144 cm and the results are almost the same in the y direction. The emittance growth can be further reduced by the abovementioned matching techniques [26,34], while the prerequisite is the suppression of the evolution or head erosion of the driver such as in our example, since the variation of the drive beam changes the plasma wake and the corresponding focusing force for $e^{+}$ beam.

The evolution of $\langle E_{z}\rangle$ for $e^{+}$ beam is depicted in Fig. 3D, exhibiting little change from the propagation distance of 20 to 80 cm. However, $\langle E_{z}\rangle$ is notably lower for the beam head at s= 140 cm, which also indicates the evolution of the drive beam and is a signal of the pump depletion. The mean energy of the positron beam increases linearly during acceleration as shown in Fig. 3E. After 144 cm propagation, the $e^{+}$ beam is accelerated to 4.91 GeV, corresponding to a mean gradient of 1.64 GeV/m, with final energy spread ΔE/E= 1.27% where ΔE is the rms of the beam energy. The final longitudinal phasespace for the positron beam (Fig. 3F) is consistent with the structure of $E_{z}$. The final spectra (Fig. 3G) show nearly complete energy depletion of some drive electrons, and energy transfer efficiency from the wake to the positron beam $\eta =Q_{p}E_{p}^{+}/Q_{e}E_{e}^{-}$ is 25.5%.

**Fig. 4.**
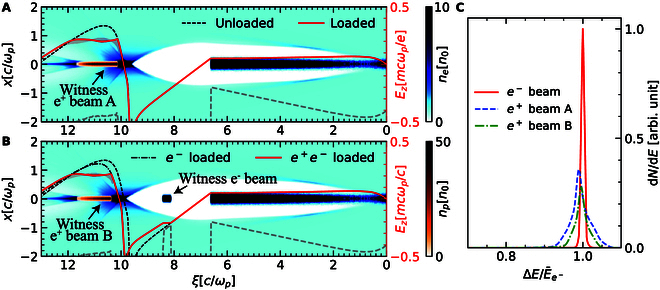
Configurations of the HTR (A) positron and (B) simultaneous electron and positron acceleration. Gray dashed lines denote the beam current profile. (C) Spectra of the accelerated electron and positron beams.

Besides, high-quality positron beam acceleration in the blowout regime opens new possibilities for advanced accelerator concepts. HTR acceleration enables the witness beam to gain energy multiple times that of the initial energy of the drive beam, making it crucial for high energy applications [37]. Previously, relevant discussions were focused on the plasma wakefield electron acceleration, whereas HTR acceleration now can be directly applied to positron beam acceleration. In Fig. 4A, we present a case of HTR positron acceleration in a customized $e^{-}$ beam-driven blowout regime. The plasma density is the same as above, and the drive beam has a transverse size 5 μm and current profile $I\left(\xi \right)\propto 2k_{p}\xi -1+5e^{-2k_{p}\xi }$ with total length 400 μm and charge 1.4 nC. Assuming nonevolving beams, an optimized $e^{+}$ beam obtained through the slice-by-slice optimized procedure is presented. The positron beam has transverse size 2 μm, contains 131 pC charge, and experiences an average accelerating field of 1.78 GV/m that is 3 times the maximum decelerating field of the driver. With an initial energy of 10 GeV for the drive and witness beams, the positron beam can be accelerated to about 40 GeV in this regime. The induced rms energy spread for the positron beam is 2.75% and the beam load energy transfer efficiency is 35.3%.

Furthermore, an electron beam can also be loaded in the first bucket of the same wake to achieve simultaneously HTR $e^{+}e^{-}$ acceleration as in Fig. 4B. The witness $e^{-}$ and $e^{+}$ beams contain similar charge and obtain the same amplitude of accelerating gradient. By loading an extra electron beam, the energy spread for the positron beam is improved due to less intense loaded wake while the overall energy transfer efficiency 44.1% is increased compared to the single witness beam case as listed in Table. The transverse parameters of the $e^{-}$ beam have a relatively large space and the matched $e^{+}$ beam emittance is several mm·mrad. The spectra for the witness beams are plotted in Fig. 4C. Compared with the $e^{-}$ beam, $e^{+}$ beams obtain a greater energy spread due to their strong coupling of the plasma electron filaments. The simultaneous acceleration of the $e^{-}$ and $e^{+}$ beams with similar parameters provides a novel approach for future high-energy particle physics.

**Table.** Characteristics of the HTR positron acceleration

Witness beam	Q (pC)	$q\bar{E}$ (GeV/m)	$\Delta_{\mathrm{rms}}\;$(%)	η (%)
$e^{+}$ beam A	131	1.78	2.75	35.3
$e^{+}$ beam B	80.4	1.78	2.03	21.3
$e^{-}$ beam	84.0	1.78	0.29	22.8

## Discussion

We have analyzed the positron beam loading in the blowout regime via PIC simulations and a phenomenological theoretical model and demonstrated its capability for high-quality positron acceleration with tens of percent energy transfer efficiency from wake to witness beam. The physics of positron beam loading is strongly determined by the dynamics of plasma electrons that can form an electron filament that overlaps with the positrons, and the beam quality is sensitive to the distribution of electrons in the filament. We have further shown that it is possible to simultaneously beam load an electron and a positron beam in a nonlinear blowout wake driven by an electron beam, and to obtain high gradient acceleration and efficiency acceleration of both beams. These ideas may be applied to laser-driven nonlinear wakes as well. Further refinement of the theoretical model is also possible, which will lead to further improvement in the prospects of positron beam acceleration in plasma accelerators.

However, the positron beam loading theory proposed in this study is not self-consistent, as the structure of the electron filament and shape of the second blowout cavity cannot be derived from the parameters of the loaded positron beam. One missing aspect is the specific dynamics of the sheath electrons, particularly their behavior when they return to the axis. Within this region, the conventional theory of blowout regime becomes less effective, and incorporating multiple sheath components yields a more refined description [38]. Nevertheless, it fails to capture the intricate dynamics occurring at the axis—the singularity of the blowout regime. Therefore, this study focuses on the essential change in the bubble structure induced by positron beam loading, as well as the potential for achieving high-quality positron acceleration. These phenomenological theoretical findings are consistent with the results of PIC simulations, although a self-consistent study of positron beam loading will require a more detailed investigation of the blowout regime. In addition, in all simulations presented in this work, the plasma is assumed to be initially cold. When the plasma has a finite temperature, the fine structure of the positron beam loading-induced electron filament may be modified accordingly, which could potentially benefit both the beam quality and numerical convergence in simulations [39].

The findings in this work are of considerable significance for applying PWFAs into high-energy physics. For linear electron–positron colliders, the preferred beam parameters are on the order of ~nC in bunch charge, normalized emittances of 10 and 0.01 mm·mrad in the horizontal and vertical planes, respectively, and an energy spread below 1% [40]. Assuming ideal manipulation of the transverse phasespace, a round beam accelerated in plasma would require an emittance less than 1 mm·mrad. The beam parameters obtained in this study—in terms of charge, emittance, and energy spread—are generally within one order of magnitude of the desired values. Higher positron bunch charges can be achieved by operating at lower plasma densities. However, simultaneously reducing the emittance would necessitate a much smaller matched beam size throughout the acceleration process. Based on previous studies under similar conditions [25], emittance growth can remain well controlled even in strongly nonlinear focusing fields, provided the focusing force is relatively stable. Nevertheless, this would require more advanced simulation tools, such as mesh refinement techniques [41], to obtain reliable predictions under these scaled conditions. Regarding energy spread, a key challenge is to reduce it while maintaining high beam loading efficiency. Preliminary studies suggest that incorporating finite plasma temperature may help mitigate the energy spread of the loaded positron bunch [39]. Further definitive conclusions will require in-depth investigations into sheath electron dynamics and interactions between plasma electrons and positron beam. In addition, recently proposed and demonstrated energy compression and stabilized schemes could be applied to achieve the desired energy spread [42], especially in scenarios with relaxed bunch length constraints.

Furthermore, for an injector in next-generation colliders where plasma-accelerated electron and positron beams are injected into a booster or collider ring, the beam parameter requirements are more relaxed compared to linear colliders. As specified in the CEPC Conceptual Design Report and Technical Design Report, the booster injection requirements include a beam charge of ~1 nC, transverse emittances of several hundred mm·mrad, and an energy spread of approximately 0.2% [6]. By scaling to lower plasma densities, the presented results can approach the required charge and emittance. Further combined with some energy spread compression techniques, the energy spread can potentially be reduced by an additional order of magnitude [42], thereby enabling the beam to meet the primary injector requirements. These insights carry substantial implications for the development of future particle colliders.

## Methods

### High-fidelity PIC simulation

Accurately modeling the plasma electron response and positron beam dynamics is crucial for plasma wakefield positron acceleration. To achieve both high numerical accuracy and computational efficiency for a long-distance acceleration, we employ the open-source 3D simulation code QuickPIC [32,33] in this work. This code leverages the QSA, where the plasma response is computed assuming the shape of the laser or particle beam (including its envelope and energy or frequency) remains static. The resulting plasma wakefields are then used to advance the laser or beam forward with a considerably larger time step. In high-energy PWFA acceleration, QSA-based simulations have shown excellent agreement with fully electromagnetic 3D simulations while improving computational efficiency by 2 to 3 orders of magnitude.

In Figs. 1 and 2, the simulation window is $14\times 14\times 15\;k_{p}^{-3}$ in $\left(x,y,\xi \right)$ with a resolution of $0.014\times 0.014\times 0.015\;k_{p}^{-3}$. For Figs. 3 and 4, the drive electron beam has a lower peak current, resulting in a smaller blowout radius. Consequently, a more compact simulation window of $6\times 6\times 15\;k_{p}^{-3}$ was chosen, allowing for a higher resolution of $0.006\times 0.006\times 0.015\;k_{p}^{-3}$. In this simulation, the rms size of the positron bunch is $0.04\;k_{p}^{-1}$, which is comparable to the scale of the on-axis electron filament. This level of resolution is sufficient to accurately capture the transverse dynamics of the positron beam. Moreover, due to the relatively low charge and peak density of the positron bunch, as well as its short bunch length, the background plasma electrons provide effective charge neutralization, allowing the plasma ions to be considered stationary throughout the simulation.

### Calculation of the pseudo-potential in a coaxial blowout cavity

For PWFA where the electron and positron beams are highly relativistic, the timescale of the beam evolution is much longer than that of plasma electrons. Through performing a coordinate transformation $\left(x,y,z,t\right)$ to $\left(x,y,\xi \equiv \mathrm{ct}-z,s\equiv z\right)$, we can reasonably assume that the plasma structure and wakefield depend on 𝜉, i.e., the QSA $\left(\partial_{s}\ll \partial_{\xi }\right)$. Under the QSA, Eq. 4 can be derived from the Maxwell equations where $\psi \equiv \varphi -A_{z}$ is the pseudo-potential. Furthermore, the phenomenon under investigation can be considered axisymmetric. Thus, knowing the radial distribution of $\rho -J_{z}/c$ is sufficient to determine ψ.

To obtain Eq. 5, the continuity equation of charge under QSA

$$\frac{d}{\mathrm{d\xi}}\int_{0}^{+\infty }r\left(\rho -J_{z}/c\right)\mathrm{dr}=0$$ (8)

should also be employed. In front of the drive beam, the plasma is unperturbed, so $\int r\left(\rho -J_{z}/c\right)\mathrm{dr}=0$ at every slice. At the positron beam loading region, it gives

$$\left(n_{e}-1\right)r_{e}^{2}-\left(r_{b}^{2}-r_{e}^{2}\right)+n_{\Delta }\left[{\left(r_{b}+\Delta \right)}^{2}-r_{b}^{2}\right]=0.$$ (9)

The on-axis pseudo-potential $\begin{array}{c} \psi_{0}\left(\xi \right)=\frac{1}{4}r_{b}^{2}\left(\xi \right)\left(1+\beta \left(\xi \right)\right)-\frac{1}{4} \end{array}$$\begin{array}{c} n_{e}\left(\xi \right)r_{e}^{2}\left(\xi \right)\left(1+\beta \left(\xi \right)\right)-\frac{1}{2}n_{e}\left(\xi \right)r_{e}^{2}\left(\xi \right)\text{ln}\frac{r_{b}\left(\xi \right)}{r_{e}\left(\xi \right)} \end{array}$ can be obtained by solving Eq. 4 and then simplified with Eq. 9. It is evident that this potential term has 3 distinct contributions: the boundary of the blowout cavity, the on-axis electron filament, and the coupling between the electron filament and the sheath boundary.

### Optimization of the positron beam loading current profile

An important objective of beam loading research is to optimize parameters for efficient, high-quality acceleration. For electron acceleration in the blowout regime, theoretical works have already provided reliable predictions of the beam current profile. However, for positron beam loading, there is no established theory to describe the specific impact of beam parameters on the plasma wakefield as mentioned above. Current optimization primarily relies on high-precision numerical simulations. Since the loaded positron bunch is ultra-relativistic, causality ensures that each beam slice only affects the wakefield behind it. Therefore, an iterative algorithm that shares a concept similar to that in Ref. [26] can be designed to compute the required current profile from head to tail.

The steps of the algorithm are as follows: The positron loading region is longitudinally divided into *n* slices, which are traversed sequentially from head to tail. For any slice *i*, the initial charge in the slice is set to zero, and the weighted average field strength $\left\langle E_{z}\right\rangle$ within the slice is calculated and compared with the target accelerating field $E_{0}$. If $\left\langle E_{z}\right\rangle \leq E_{0}$, the computation for this slice terminates and if $\left\langle E_{z}\right\rangle >E_{0}$, an iterative process begins. For the beginning of the iterative process, the charge in the slice is increased by a fixed increment *q*, and $\left\langle E_{z}\right\rangle$ is recalculated. For the other iterative step, if $\left\langle E_{z}\right\rangle >E_{0}$, the charge is increased again by *q*, while if $\left\langle E_{z}\right\rangle <E_{0}$, the charge is set to the average of the current and previous iteration’s charge. The iteration terminates when $|\left\langle E_{z}\right\rangle -E_{0}|<{\varepsilon E}_{0}$, where ε is the predefined error tolerance (typically much smaller than the slice energy spread).

To prevent sampling bias due to insufficient or uneven particle distribution in the slice—and because the initial charge of zero would make it impossible to compute the weighted average field for the positron beam—a test beam can be introduced in the positron loading region. This test beam should have the same transverse distribution as the loaded bunch, a sufficiently large number of particles, and a sufficiently small charge. The weighted average $\left\langle E_{z}\right\rangle$ in subsequent iterations can then be calculated using this test beam.

To further accelerate the convergence of the iteration for each slice, adaptive step size methods can be employed.

## Data Availability

The data that support the plots within this paper and other findings of this study are available from the corresponding author upon reasonable request.
